# Blood Being Tricky: Anticoagulation-Resistant Venous Thromboembolism (VTE)

**DOI:** 10.7759/cureus.25914

**Published:** 2022-06-13

**Authors:** Raghu Tiperneni, Adhithya Rajamohan, Rana Prathap P Padappayil, Vishakha Sirpal, Harshil Fichadiya

**Affiliations:** 1 Internal Medicine, Rutgers Health/Monmouth Medical Center, Long Branch, USA

**Keywords:** pulmonary embolism (pe), ekos catheter, anticoagulation resistance, deep vein thrombosis (dvt), venous thromboembolism (vte)

## Abstract

Venous thromboembolism (VTE) is a condition in which blood clots form in the venous system of the body. It includes deep venous thrombosis (DVT) that occurs when a blood clot forms in a deep vein, more common in lower extremities, and pulmonary embolism (PE) as the clot breaks loose and travels through the bloodstream to the pulmonary arteries. VTE can result in significant morbidity and mortality. It is a preventable medical condition with the treatment being straightforward in most cases with anticoagulation and mechanical thrombectomy. Here, we discuss a rare case of a 40-year-old male with recurrent VTE that was resistant to different available therapeutic options such as direct oral anticoagulants (DOAC), vitamin k antagonists, heparin, and catheter-directed thrombolysis presenting with occlusive thrombus in the entirety of the right-sided deep venous structures, with minimal preservation of flow in the common femoral vein.

## Introduction

Venous thromboembolism (VTE) is a condition in which blood clots form in the venous system of the body. It includes deep venous thrombosis (DVT) that occurs when a blood clot forms in a deep vein, more common in lower extremities, and pulmonary embolism (PE) as the clot breaks loose and travels through the bloodstream to the pulmonary arteries. VTE can result in significant morbidity and mortality. The treatment is straightforward in most cases with anticoagulation and mechanical thrombectomy. The post-thrombotic syndrome has been estimated to affect 23%-60% of individuals with DVT, frequently occurring within two years of the DVT episode [1]. PTS symptoms include chronic leg pain, swelling, redness, and ulcers (sores) [2]. Risk factors include ipsilateral DVT recurrence, persistent venous symptoms, and signs one month after acute DVT, residual thrombosis on ultrasound, and persistent elevation of d-dimer [3].

## Case presentation

The patient is a 40-year-old male with a medical history of recurrent VTE, s/p inferior vena cava filter after bilateral PE, middle cerebral artery stroke presented in November 2020 with right lower extremity pain, swelling, and is on enoxaparin with reported compliance. The patient’s previous admissions for VTE and received treatment are outlined in Table 1. In April 2019, the patient also underwent a hypercoagulable workup including factor V Leiden mutation, antithrombin III mutation, and lupus anticoagulant, which were all negative. The workup for underlying malignancy with colonoscopy, imaging, and blood work was negative.

**Table 1:** Patient’s previous admissions for VTE and received treatment DVT: Deep venous thrombosis; IVC: Inferior vena cava; PE: pulmonary embolism; EKOS: EkoSonic endovascular system; VTE: Venous thromboembolism.

Admission	Diagnosis	Treatment received	Anticoagulant at discharge
Early March 2019	DVT of left popliteal vein	Eliquis, 5 mg twice daily	Eliquis, 5 mg twice daily
Late March 2019	DVT extension to the left distal femoral vein	Eliquis, 5 mg twice daily	Eliquis, 5 mg twice daily
April 2019	Progression to completely occlusive DVT from left popliteal vein to the mid femoral vein with slight compression of common iliac vein	Dabigatran, 150 mg twice daily	Dabigatran, 150 mg twice daily
June 2019	Bilateral PE	IVC filter placement	Dabigatran, 150 mg twice daily
August 2019	Worsening DVT extending into left posterior tibial and peroneal veins	Warfarin 5 mg daily (adjusted as per INR)	Warfarin 5 mg daily
September 2019	New right-sided common vein DVT and clotted IVC filter to the level of bifurcation (on therapeutic INR 3.5)	EKOS catheter placement, with alteplase and heparin infusion treatment, subsequently failed EKOS catheter treatment with minimal improvement in clot size. Then, he underwent a vacuum clot suction.	Therapeutic enoxaparin 60 mg subcutaneous twice daily

**Figure 1:**
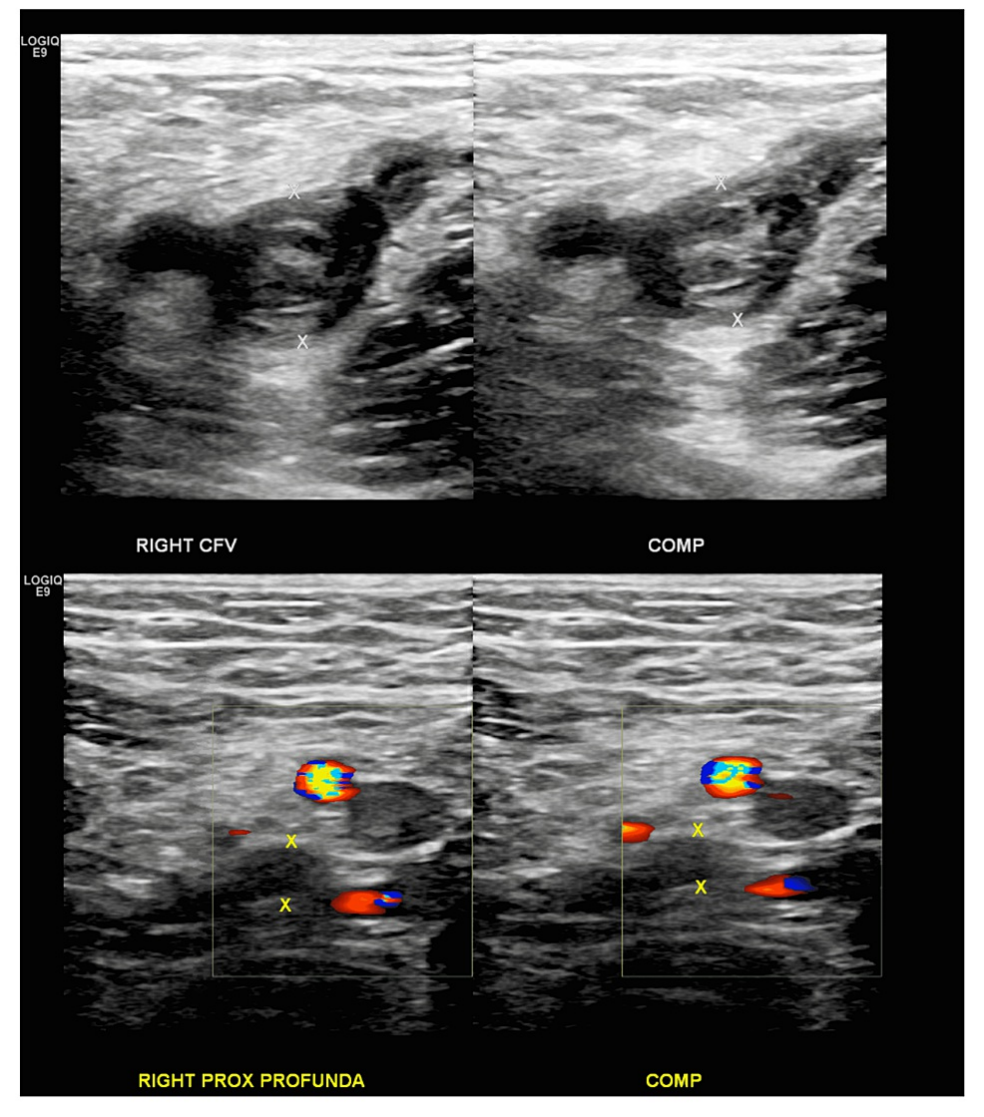
Right lower extremity Doppler ultrasound showing extensive occlusive thrombus in the entirety of the right-sided deep venous structures, with minimal preservation of flow in the common femoral vein CFV: Common femoral vein.

Vitals on his admission were stable with a blood pressure of 110/70 mmHg, heart rate of 79 beats/min, on room air saturating 98%. Labs revealed normal hemoglobin at 12.2 g/dl, platelet at 322,000 per microliter of blood, INR at 1, PT at 10.1 seconds, and partial thromboplastin time (PTT) at 63.7 seconds. Imaging with lower extremity Doppler showed extensive occlusive thrombus in the entirety of the right-sided deep venous structures, with minimal preservation of flow in the common femoral (Figure 1).

**Figure 2:**
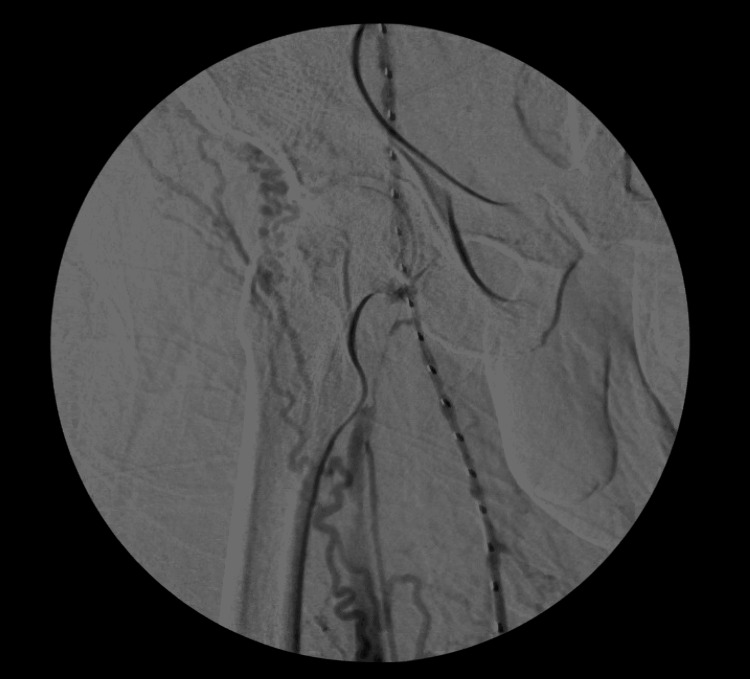
EkoSonic endovascular system (EKOS) catheter

Computed tomography angiography of the lungs was negative for PE. Given the extensive occlusive thrombus, vascular surgery was consulted. He was started on a heparin drip, and an EkoSonic endovascular system (EKOS) catheter was placed again with an alteplase infusion (Figure 2).

**Figure 3:**
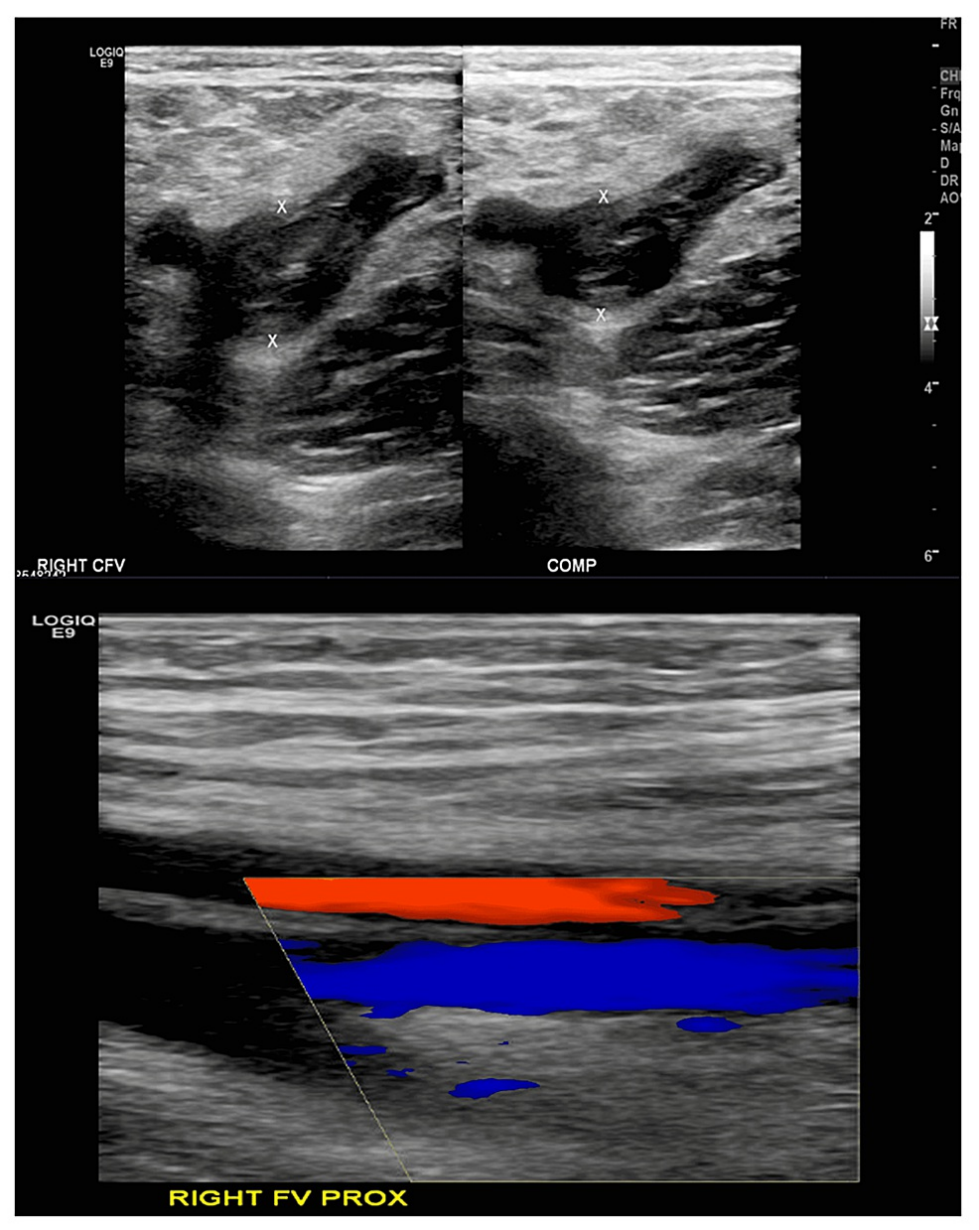
Main venous system with collaterals displaying better flow to the anterior right lower extremity on contrast study CFV: Common femoral vein; FV: Femoral vein.

The EKOS catheter was removed four days later. At this point, chronic clots were observed in the main venous system with collaterals displaying better flow to the anterior right lower extremity in contrast study (Figure 3).

The chronic swelling and pain due to chronic clots' venous insufficiency are from the back pressure. He was discharged on therapeutic enoxaparin 60 mg subcutaneously twice daily. He had further hospitalizations in November 2020, December 2020, and January 2021 for right thigh pain attributed to post-thrombotic syndrome and cellulitis.

## Discussion

Indefinite anticoagulation therapy, rather than three months, is recommended for patients with recurrent DVT or symptomatic PE without identifiable risk factors (unproved VTE) and with low bleeding risk. For those with high bleeding risk, the benefits of indefinite anticoagulant therapy are less certain and depend heavily on patient-specific thrombotic and bleeding risk as well as the patient's values and preferences [3,4].

The recommendation for indefinite anticoagulation in patients with more than one episode of unprovoked DVT or symptomatic PE is based upon recurrence rates that are approximately 15% at one year and 45% at five years. One meta-analysis of five randomized trials of patients with VTE reported that extending therapy in patients with a second unprovoked VTE event resulted in 132 and 396 fewer events per 1000 patients at one year and five years, respectively, compared with a three- or six-month period of anticoagulation [5].

The duration of the anticoagulant trial study has shown that no patient developed a recurrent VTE when on warfarin for a four-year period. This raises the question of why there is recurrent VTE for this patient (despite no underlying causes). DVT while on anticoagulation poses a difficult scenario to navigate and manage for physicians including hematologists. The logical explanation in most cases is medication nonadherence.

Post-thrombotic syndrome (PTS) is a common complication in almost half of the patients with a DVT. PTS can cause severe symptoms that it is often confused with a presentation of a recurrent DVT. It is hence important to treat PTS to prevent further admissions under the misdiagnosis of recurrent DVT. Some important treatment options are elastic compression stockings, pneumatic compression devices if symptoms are not controlled on endoscopic ultrasound (EUS), and in some cases, graded exercise programs [5,6]. The PTS has a significant impact on the quality of life. Further research is needed to assess the value of including quality of life as a routine measure of outcome in clinical studies of patients with DVT and PTS [7,8].

## Conclusions

Indefinite anticoagulation therapy, rather than three months, is recommended for patients with recurrent DVT or symptomatic PE without identifiable risk factors (unproved VTE) and with low bleeding risk. For those with high bleeding risk, the benefits of indefinite anticoagulant therapy are less certain and depend heavily on patient-specific thrombotic and bleeding risks as well as the patient's values and preferences. Despite indefinite anticoagulation, there might be cases of recurrent VTE; therefore, we need further research to identify the treatment modalities in such cases. As of now, we have similar treatment modalities for recurrent and non-recurrent VTE.

PTS is a common complication in almost half of the patients with a DVT. PTS can cause severe symptoms, so it is often confused with a recurrent DVT presentation and has an impact on the quality of life. Hence, it is important to treat PTS to prevent further admissions under the misdiagnosis of recurrent DVT.
